# Limited added value of fungal ITS amplicon sequencing in the study of bovine abortion

**DOI:** 10.1016/j.heliyon.2018.e00915

**Published:** 2018-11-05

**Authors:** Sara Vidal, Bernd W. Brandt, Martina Dettwiler, Carlos Abril, Jenny Bressan, Gilbert Greub, Caroline F. Frey, Vincent Perreten, Sabrina Rodriguez-Campos

**Affiliations:** a Institute of Veterinary Bacteriology, Vetsuisse Faculty, University of Bern, Laenggassstrasse 122, CH-3012 Bern, Switzerland; b Department of Preventive Dentistry, Academic Centre for Dentistry Amsterdam, University of Amsterdam and VU University Amsterdam, Gustav Mahlerlaan 3004, 1081 LA Amsterdam, the Netherlands; c Institute of Animal Pathology, Vetsuisse Faculty, University of Bern, Laenggassstrasse 122, CH-3012 Bern, Switzerland; d Institute of Virology and Immunology, University of Bern, Laenggassstrasse 122, CH-3012 Bern, Switzerland; e Department of Neurology, Bern University Hospital and University of Bern, Freiburgstrasse, CH-3010 Bern, Switzerland; f Institute of Microbiology, University Hospital Center and University of Lausanne, Bugnon 48, CH-1011 Lausanne, Switzerland; g Institute of Parasitology, Vetsuisse Faculty, University of Bern, Laenggassstrasse 122, CH-3012 Bern, Switzerland

**Keywords:** Microbiology, Veterinary science, Infectious disease

## Abstract

Bovine mycotic abortion is sporadic and caused by different ubiquitous and opportunistic fungi. Recently, a broad spectrum of bacterial opportunists involved in bovine abortion was revealed by 16S rRNA gene amplicon sequencing. We hypothesized that fungal organisms potentially involved in bovine abortion also might remain undetected by conventional culture. In this retrospective study, we therefore applied fungal internal transcribed spacer 2 (ITS2) region amplicon sequencing to 74 cases of bovine abortion submitted to our diagnostic service. The investigation was complemented by fungal culture and, retrospectively, by data from bacteriological, virological and parasitological analyses and histopathological examination of placentas.

Fungal DNA was found in both the placentas and abomasal contents, with 92 fungal genera identified. In 18 cases, >75% of the reads belonged to one specific fungal genus: *Candida* (n = 7), *Malassezia* (n = 4), *Cryptococcus* (n = 3), unidentified *Capnodiales* (n = 3), *Actinomucor* (n = 1), *Cystofilobasidium* (n = 1), *Penicillium* (n = 1), *Verticillum* (n = 1) and *Zymoseptoria* (n = 1) with one case harboring two different genera. By culture, in contrast, fungal agents were detected in only 6 cases. Inflammatory and/or necrotizing lesions were found in 27/40 histologically assessed placentas. However, no lesion-associated fungal structures were detected in HE- and PAS-stained specimens. Complementary data revealed the presence of one or more non-fungal possible abortifacient: *Chlamydiales*, *Coxiella burnetii*, *Leptospira* spp., *Campylobacter fetus* subsp. *fetus*, *Streptococcus uberis*, *Escherichia coli*, *Streptococcus pluranimalium*, *Bacillus licheniformis*, *Campylobacter fetus* subsp. *fetus*, *Serratia marcescens*, *Trueperella pyogenes*, Schmallenbergvirus, *Neospora caninum*. The mycobiota revealed by sequencing did not differ between cases with or without a possible infectious etiology.

Our study suggests that amplicon sequencing of the ITS2 region from DNA isolated from bovine abortion does not provide additional information or new insight into mycotic abortion and without complementary analyses may easily lead to a false interpretation of the role of fungal organisms in bovine abortion.

## 1 Introduction

Abortion in cattle causes significant economic losses and requires prompt diagnosis of possible causative agents (Hässig et al., 2000). The causes of abortion are diverse and involve multiple, partly zoonotic, etiologic agents such as viruses, bacteria, fungi and parasites (Murray, 1990; Cabell, 2007; Barkallah et al., 2014). Mycotic abortion is sporadic and usually occurs in the third trimester of pregnancy (Walker, 2007; Austin, 2015; Pal, 2015). In cows, the global prevalence of mycotic abortion ranges from 2 to 20%, depending on the environment, the location and the time of the year; as it typically affects less than 10% of a herd, outbreaks are unlikely (Austin, 2015; Pal, 2015). Most of the fungi involved in abortion, including filamentous molds and unicellular yeasts, are saprophytes, which are found in moist organic environments.

Among these fungi, *Aspergillus fumigatus* has been identified as the etiological agent in approximately 60–80% of cases, followed by members of the class *Zygomycetes* (21% of cases). Other species of fungi that have also been associated with bovine abortion are *Lichtheimia* spp., *Rhizopus* spp., *Mortierella* spp., *Mucor* spp., and yeast of the genus *Candida* (Knudtson and Kirkbride, 1992; Walker, 2007; Pal, 2015). Fungal organisms are thought to colonize a pregnant uterus mainly through hematogenous spread as a consequence of pulmonary or gastrointestinal mycotic infections. Primary ascending infections from the lower urogenital tract are rather rare, and co-infections are more common (Schlafer and Foster, 2016). The gold standard workflow for accurate diagnosis of mycotic abortion includes (i) microscopic visualization of fungal hyphae and elements in placenta or fetal tissues and fluids with compatible gross lesions and (ii) detection of fungi or fungal DNA in the abortion material via culture or molecular methods (Kirkbride, 1990; Walker, 2007; Austin, 2015).

Regardless, this workflow should always be combined with detection of other abortifacients, including viruses, bacteria and parasites. Viral agents include bovine herpesvirus type-1 (BHV-1), bovine viral diarrhea virus (BVDV) (Kelling, 2007; Anderson, 2012; Baumgartner, 2015) and, sporadically, Schmallenberg virus (SBV) and Bluetongue virus (Borel et al., 2014). The most common bacterial abortifacients of cattle include zoonotic pathogens such as *Brucella* spp., *Coxiella burnetii* and *Chlamydia abortus* (Yaeger and Holler, 2007; Anderson, 2012; Borel et al., 2014). Moreover, new evidence implicates other *Chlamydia*-related bacteria (Borel et al., 2007; Deuchande et al., 2010; Barkallah et al., 2014; Vidal et al., 2017a) as well as other zoonotic bacteria such as *Salmonella* spp., *Campylobacter* spp., *Leptospira* spp. and *Listeria monocytogenes* (Yaeger and Holler, 2007; Borel et al., 2014). Among parasitological causes, protozoal infection with *Neospora caninum* represents the most frequent cause of bovine abortion worldwide, with a prevalence of up to 40%, and such organisms therefore need to be included in differential diagnosis (Abbitt and Rae, 2007; Estill and Scully, 2015).

Recently, 16S rRNA gene amplicon sequencing was found to be useful for identifying a wide spectrum of pathogens from bovine abortion material, providing insight into new species and polymicrobial infections (Vidal et al., 2017b). Due to the considerable limitations in detecting mycobiota by culture methods, the number of studies investigating fungal microbiota using amplicon sequencing of the ribosomal internal transcribed spacer (ITS) region, the universal DNA barcode marker for fungi, is increasing (Schoch et al., 2012; Zoll et al., 2016). However, no studies have specifically investigated the presence of fungal microbiota in abortion material.

We hypothesized that uncultured fungi might be present in bovine abortion material and might play a role in multi-infections. The aim of this study was to apply next-generation sequencing (NGS) of the ITS2 region to identify the mycobiota present in abortion material and thus to possibly uncover novel fungi overlooked in routine diagnosis. Retrospectively, we gathered additional diagnostic data for the detection of bacterial, viral and parasitical abortifacients and histopathological examination of the placenta, if available, was conducted to place the sequencing results within the context of broad-spectrum analyses of these abortions.

## 2 Material and methods

### 2.1 Collection of samples

Samples from 74 cases of bovine abortion (Supplementary Table 1) from Central and Western Switzerland submitted for routine abortion investigation between October 2012 and March 2015 were included in the study. Veterinarians obtained the samples for diagnostic purposes and/or to check the health status of the cattle population. Neither action requires ethical approval or a permit for animal experimentation according to the current Swiss legislation [Federal Animal Protection Law, 455, Article 3 Paragraph c (https://www.admin.ch/opc/de/classified-compilation/20022103/index.html)]. This work is in line with the European legislation (Directive 2010/63/EU of the European Parliament and of the Council, Article 40).

The samples originated from the following Swiss cantons: Bern, n = 44; Fribourg, n = 7; Vaud, n = 5; Luzern, n = 4; Solothurn, n = 4; Aargau, n = 3; Basel-Land, n = 3; Jura, n = 3; and Valais, n = 1. The canton Bern has the highest number of cattle in Switzerland with approx. 20% of the 1.5 million head of cattle and 28% of the 1.1 million head of cattle in Central and Western Switzerland (https://www.agate.ch/portal/web/agate/die-tierverkehrsdatenbank-tvd, consulted on May 9, 2018). The month of gestation was used to assign the cases to three trimesters: in 64 cases the abortion occurred in the last trimester, in four cases in the second trimester and in six cases the month of gestation was unknown. Among all cases, 59 submissions were complete [entire fetus and placenta (PL)]; in the remaining cases, the placenta and organs from the fetus were not available for all the analyses (Supplementary Table 1). Necropsies of the complete submissions were performed according to standard protocols and placenta, fetal abomasum, liver and lung were forwarded for further laboratory analyses. The number of analyses depended on the suitability and availability of the sample for a specific approach and/or whether the analyses were requested by the submitter. Maternal serum was also received for 63 cases. Samples were obtained according to Vidal et al. (2017b). As negative controls for amplicon sequencing, we included the healthy PL, fetal abomasal content (AC) and amniotic fluid (AF) obtained from cows in calf that died for non-infectious reasons.

### 2.2 Total DNA extraction, Illumina MiSeq sequencing and sequence data processing

Extraction of total genomic DNA was performed for all samples of PL (n = 72) and AC (n = 63) submitted and from healthy fetuses [PL (n = 3), AC (n = 3) and AF (n = 3)] according to Vidal et al. (2017a; 2017b). DNA extraction included two extraction control tubes containing only reagents.

Classic polymerase chain reaction (PCR) amplification of the fungal ITS2 region was performed to verify the presence of fungal DNA before sequencing. The primers used were ITS3 (5′- GCA TCG ATG AAG AAC GCA GC -3′) and ITS4 (5′- TCC TCC GCT TAT TGA TAT GC -3′) (Kumar and Shukla, 2005). PCR was performed in 30 μL reaction mixtures containing 1x PCR buffer, 2 mM MgCl_2_, 0.4 μM forward and reverse primer, 200 μM deoxyribonucleotide triphosphates (dNTPs), 0.25 μL of 5 U/μL thermostable DNA FIREPol® Polymerase Solis BioDyne, 21.25 μL of sterile water and 2 μL of DNA solution. The following conditions were applied: 94 °C for 3 min, followed by 35 cycles of 95 °C for 30 s, 50 °C for 30 s, and 72 °C for 1 min, and a final elongation step at 72 °C for 8 min. The two extraction control tubes were included.

Sequencing was performed at Microsynth (Balgach, Switzerland) for 74 cases [PL (n = 55); AC (n = 40)]. The ITS2 region from the fungal internal transcribed spacer was sequenced, using the two-step PCR protocol (cf Illumina Part # 15044223 Rev. B) with ITS3 and ITS4 as locus-specific primers. Subsequently, the PCR libraries were mixed in equimolar ratios and sequenced using the Illumina MiSeq platform and MiSeq Reagent Kit v3 (600 cycle kit).

Sequencing reads were merged (Edgar and Flyvbjerg, 2015), processed, and clustered using USEARCH version 8.0.1623 (Edgar, 2013). The ITS sequence data were first filtered to remove reads shorter than 80 nt and reads with ambiguous bases, after which the reads were screened for the presence of the ITS primer, allowing one mismatch. Read pairs containing the forward and reverse ITS primer one time were then merged after removal of the primers, allowing staggered alignments and a length between 70 and 590 nt. The merged read pairs were quality filtered (max. expected error rate 0.002), and the remaining merged reads were checked for the presence of PhiX and chimeric (uchime_denovo) sequences. The fungal ITS2 region was extracted from all sequences (to which the used ITS primers were reattached) using ITSx version 1.0.11 (Bengtsson-Palme et al., 2013) with fungal models only and default parameters otherwise. Only ITS2 regions originating from quality-filtered and chimeric-free sequences were clustered at a 97% identity level into operational taxonomic units (OTUs) in line with the UPARSE pipeline (Edgar, 2013) using the following adaptations: –uparse_maxdball 1200, only de novo chimera checking, and usearch_global with –maxaccepts 8 –maxrejects 64 –maxhits 1. During the global alignment step, all extracted ITS2 regions were mapped to OTU centroids. The most abundant sequence of each OTU was selected using QIIME version 1.8.0 (Caporaso et al., 2010) and assigned a taxonomical classification using the Ribosomal Database Project (RDP) classifier (Cole et al., 2009) with a minimum confidence of 0.8 and the ITS2 regions extracted by ITSx from the UNITE dataset (QIIME release, version 7.0, dynamic use of clustering thresholds; file: sh_refs_qiime_ver7_dynamic_s_31.01.2016.fasta) (Kõljalg et al., 2013). The sequencing depth was normalized by sub-sampling the dataset from the BIOM table randomly to 1,000 reads per sample. Bacterial distributions at the phylum, class, order, family and genus levels were summarized to group the samples by organ (PL or AC) and plotted using the script summarize_taxa_through_plots.py in QIIME 1.9.1 (Caporaso et al., 2010).

### 2.3 Broad-spectrum bacterial and fungal culture

For all samples, including those from control fetuses [PL (n = 75), AC (n = 66), AF (n = 3), liver (n = 61), lung (n = 61)], broad-spectrum bacterial and fungal culture was performed according to Schnydrig et al. (2017).

### 2.4 BHV-1, BVDV and SBV

Detection of antibodies against BHV-1 was carried out for 62 serum samples of dams using commercial IDEXX IBR gB X3 Ab Test (IDEXX, Liebefeld-Bern, Switzerland) according to the manufacturer's instructions.

Enzyme-linked immunosorbent assay (ELISA) for BVDV antigen detection was performed in 51 of the 74 cases using fetal ear-notch samples according to Hilbe et al. (2007).

In total, 43 cases were tested for SBV using two different methods: (i) detection of antibodies in fetal body fluids with ID Screen® Schmallenberg virus Competition Multi-species ELISA (IDVet Grabels, France), (ii) detection of viral RNA in the brain stem by quantitative reverse transcription PCR (RT-qPCR) according Bilk et al. (2012). In 12 cases, the spleen and AC were also checked for SBV.

### 2.5 *Chlamydiales*, *C. burnetii* and pathogenic *Leptospira* spp.

Molecular detection of *Chlamydiales*, *C. burnetii* and pathogenic *Leptospira* spp. was performed in all samples of PL (n = 72) and AC (n = 63) submitted using the total genomic DNA obtained for Illumina MiSeq sequencing.

Real-time PCR targeting the 16S rRNA gene of *Chlamydiales* was performed similarly to Lienard et al. (2011) using a total volume of 25 μL, 1X final concentration of TaqMan Universal PCR Master Mix (Applied Biosystems, Foster City, CA, USA), 1 μM of each primer, 80 nM of the probe, 0.5x of internal positive control (IPC) template, 0.5x IPC Mix and 2.5 μL of the template. The following conditions were applied: 94 °C for 2 min, 45 cycles of 94 °C for 15 s and 60 °C for 30 s. Amplification was performed in duplicate using a TaqMan 7500 Fast Real-time PCR System (Applied Biosystems, Zug, Switzerland). *C. abortus* DNA and water were used as positive and negative controls, respectively. Samples were considered positive when showing an exponential amplification curve up to cycle 40 in both replicates. Samples exhibiting a cycle threshold (Ct) of ≤35 cycles were sequenced according to Lienard et al. (2011).

Real-time PCR targeting IS*1111* of *C. burnetii* and *lipL*32 of *Leptospira* spp. was performed according to Vidal et al. (2017a). For 35 of the 74 cases, the results for the detection of *Chlamydiales*, *C. burnetii* and pathogenic *Leptospira* spp. were previously reported in Vidal et al. (2017a).

### 2.6 *N. caninum*

Molecular detection of *N. caninum* was performed in 60 cases. A 50 μg sample of cerebrum was homogenized and used for total genomic DNA extraction with a Qiagen tissue kit (Qiagen, Hilden, Germany) according to the manufacturer's instructions. DNA was eluted in a final volume of 200 μL. Real-time PCR targeting Nc5 of *N. caninum* was performed according to Müller et al. (2002).

### 2.7 Pathological analyses

Of the total of 74 cases, 52 were analyzed using all diagnostic methods described above. Placentas were evaluated macroscopically for color and consistency. Placentas for 40 cases were additionally examined by histopathology (12 cases presenting either fetal malformation or severe autolysis or a clear non-infectious cause of abortion were excluded from the histopathological examination). The retrospective character of the study did not allow for the additional histological assessment of fetal organs except for fetal brain in one case. The placental tissue was fixed in 10% buffered formalin for 24–48 hours and routinely embedded in paraffin. Sections (3 μm) were mounted on Thermo Scientific™ SuperFrost Plus© (Braunschweig, Germany) glass slides and stained with hematoxylin and eosin (H&E) and the Periodic Acid–Schiff (PAS) reaction. To avoid interobserver discrepancies, the slides were evaluated by one pathologist without previous knowledge of the results from microbiological analyses for the presence of inflammation, necrosis, mineralization, plant material, fungal structures and other infectious agents.

### 2.8 Statistical analysis

Population proportions with 95% confidence intervals (CIs) were calculated using NCSS 11 Statistical Software (2016) (NCSS, LLC. Kaysville, Utah, USA, ncss.com/software/ncss). Paleontological Statistics (PAST; v3.12) software (Hammer et al., 2001) was used for diversity analyses including the observed number of OTUs, Shannon Diversity Index and Chao-1. The OTU dataset was normalized by log2-transformation and data ordination by principal component analysis (PCA) and assessment of differences in microbial profiles between groups was performed by one-way PERMANOVA (Bray–Curtis similarity distance). The *p* values were corrected using Bonferroni correction; *p* < 0.05 were considered statistically significant.

## 3 Results

### 3.1 ITS2 – PCR screening

Of the 135 samples (PL, n = 72; AC, n = 63) corresponding to 74 abortions, we confirmed the presence of fungal DNA in 95 samples of abortion material (PL, n = 55; AC, n = 40). Healthy fetal samples (PL, n = 3; AC, n = 3; AF, n = 3) were all negative, and no fungal DNA was amplified in control samples, including the two negative extraction control tubes.

### 3.2 ITS2 – sequencing overview

The 95 samples positive for fungal DNA were analyzed to investigate the mycobiota. These samples were used to generate ITS2 region profiles. A total of 9,551,077 high-quality reads were obtained, with an average of 100,537.653 ± 76,904.757 sequences per sample. The number of reads per sample ranged from 373 to 296,526 (median 83,640; mean 100,537.653; SD 76,904.757). The overall number of OTUs detected was 425 based on a 97% sequence similarity threshold. After sub-sampling, 295 OTUs remained in the dataset, and three samples with fewer than 1,000 reads were excluded from further analyses.

### 3.3 Composition of fungal communities associated with AC and PL samples

Five fungal phyla were identified in the AC and PL samples (Table 1). At the class level, 18 subcategories were identified in PL and 21 in AC, with 17 shared classes. The two predominant phyla were *Ascomycota* (PL = 64%; AC = 60.4%) and *Basidiomycota* (PL = 24.7%; AC = 31.1%), accounting for 88.7% of the fungal communities in PL and 91.41% in AC (Table 1). A portion of the reads (PL = 4.8%; AC = 3.7%) could not be identified at the phylum level.

**Table 1:** Phylum-level composition of abortion material. Relative abundance of reads (%) belonging to phyla in PL (placenta) and AC (fetal abomasal content) samples.

Phylum	PL (%)	AC (%)
Unidentified Fungi	4.8	3.7
*Ascomycota*	64	60.4
*Basidiomycota*	24.7	31.1
*Chytridiomycota*	0.004	0.9
*Neocallimastigomycota*	4.3	0.2
*Zygomycota*	2.1	3.8

At the genus level, 92 taxa were observed in the samples (PL = 73; AC = 172; shared = 54); however, 19.7% of the sequences in PL and 21.8% in AC could not be identified at the genus level. The most abundant identified genus was *Cryptococcus* (PL = 10.9%; AC = 12.4%), followed by *Candida* (PL = 10.4%; AC = 10.2%) and unidentified *Capnodiales* (PL = 6.8%; AC = 11%).

In total, 19 of the 95 samples (18 cases in total) presented a high fraction of reads (≥75%) belonging to eight specific genera: *Candida* (n = 7), *Malassezia* (n = 4), *Cryptococcus* (n = 3), *Actinomucor* (n = 1), *Cystofilobasidium* (n = 1), *Penicillium* (n = 1), *Verticillum* (n = 1) and *Zymoseptoria* (n = 1) (Table 2).

**Table 2:** Comparison of samples in which ≥75% of reads belonged to a specific fungal genus. PL = placenta, AC = fetal abomasal content. The presence of hyphae or other fungal elements could not be confirmed by staining in any of the cases.

Case ID	Organ	Genera presenting ≥75% of the reads
12Ue0622	PL	*Verticillum* (84.4%)
12Ue0839	PL	*Candida* (96.9%)
12Ue0928	AC	*Malassezia* (91.8%)
12Ue1185	AC	*Malassezia* (96.7%)
12Ue1228	PL	*Cystofilobasidium* (86.3%)
12Ue1536	AC	*Actinomucor* (98.2%)
13Ue0053	PL	*Malassezia* (85.8%)
13Ue0200	AC	*Candida* (88.2%)
13Ue0236	PL	*Cryptococcus* (98.9%)
13Ue0348	PL and AC	Pl: *Candida* (90.1%); AC: *Cryptococcus* (98%)
13Ue0536	AC	*Candida* (81.9%)
13Ue0755	PL	*Cryptococcus* (90.4%)
13Ue1143	PL	*Candida* (81.1%)
13Ue1524	AC	*Malassezia* (93.2%)
13Ue1631	PL	*Penicillium* (89.3%)
14A0020	AC	*Candida* (75.1%)
14A0046	AC	*Candida* (85.3%)
15A0019	PL	*Zymoseptoria* (84.6%)

No statistically significant differences were observed between microbial profiles of placentas and abomasal contents, abortions in the second and third trimester, autolytic and not autolytic tissues, and between non-infectious and possibly infectious abortions.

### 3.4 Broad-spectrum bacterial and fungal culture

A possible abortive fungal agent was isolated by culture in only six of the 74 cases (8.1%, 95% CI [3, 16.8]) (Table 3); culturable isolates included *Aspergillus* sp. (13Ue0526Pl; 13Ue1360Pl), *Candida catenulata* (13Ue0458Pl), *C. krusei* (14A0056Pl), *C. tropicalis* (13Ue1360Pl) and unidentified yeast (13Ue1524Pl). No growth on culture media was observed for healthy fetal samples.

**Table 3:** Fungi isolated in culture and the corresponding most abundant genera found by NGS. The presence of hyphae or other fungal elements could not be confirmed by staining in any of the cases.

Sample ID	Organ	Culture	NGS
13Ue0458Pl	Placenta	*Candida catenulata*	68.7% *Mucor*; 11.6% *Candida*; 19.7% others
13Ue0526Pl	Placenta	*Aspergillus* sp.	61.5% *Cladosporium*; 37.9% *Cryptococcus*; 0.6% others
13Ue1360Pl	Placenta	*Aspergillus* sp., *C. tropicalis*	50% *Candida*; 22.9% *Debaryomyces*; 14.2% *Cystofilobasidium*; 12.9% others
13Ue1524Pl	Placenta	Unidentified yeast	58.9% *Verticillium*; 39% *Aspergillus*; 2.1% others
14A0056Pl	Placenta	*C. krusei*	60.6% *Penicillium*; 38.9% Unidentified *Chaetomiaceae*; 0.5% others
15A0009Pl	Placenta	*Aspergillus fumigatus*	84.6% Unidentified *Zymoseptoria,* 15.4% others

A possible abortive bacterial agent present in a large quantity in axenic culture was detected in 20 of the 74 cases (27.0%, 95% CI [17.4, 38.6]) (Table 4), as follows: *Streptococcus uberis* (n = 6), *Escherichia coli* (n = 6), *Streptococcus pluranimalium* (n = 4), *Bacillus licheniformis* (n = 1), *Campylobacter fetus* subsp. *fetus* (n = 1), *Serratia marcescens* (n = 1) and *Trueperella pyogenes* (n = 1).

**Table 4:** Summary of the results of different diagnostic methods indicating percentages of positive cases. BHV-1 = bovine herpesvirus type-1, BVDV = bovine viral diarrhea virus, SBV = Schmallenberg virus.

		Positives/total cases	Percentage (95% CI)
Bacterial axenic culture		20/74	27.0%, 95% CI [17.4, 38.6]
Fungal axenic culture		6/74	8.1%, 95% CI [3, 16.8]
NGS fungi (≥75%)		18/74	24.32%, 95% CI [16, 35.2]
Antibody detection in serum from dams	BHV-1	0/62	-
Antigen detection in body fluids from fetuses	SBV	11/43	25.6%, 95% CI [13.5, 41.2]
	BVDV	0/51	-
Real-time PCR	*Chlamydiales*	10/74	13.5% (95% CI [6.7, 23.5])
	*C. burnetii*	8/74	10.8% (95% CI [4.8, 20.2])
	*Leptospira* spp.	1/74	1.4% (95% CI [0, 7.3])
	*N. caninum*	9/60	15% (95% CI [7.1, 26.6])
	SBV	5/43	11.6%, (95% CI [3.9, 25.1])

CI: Confidence interval.

### 3.5 BHV-1, BVDV and SBV

All cases were negative for BHV-1 and BVDV. Of the 43 cases tested for SBV, 11 and five were positive by ELISA (25.6%, 95% CI [13.5, 41.2]) and real-time PCR (11.6%, 95% CI [3.9, 25.1]), respectively (Table 4).

### 3.6 *Chlamydiales*, *C. burnetii* and pathogenic *Leptospira* spp.

Of 74 cases tested by real-time PCR, 10 were positive for *Chlamydiales* (13.5%, 95% CI [6.7, 23.5]), eight for *C. burnetii* (10.8%, 95% CI [4.8, 20.2]) and one for *Leptospira* spp. (1.4%, 95% CI [0, 7.3]) (Table 4). One case was positive for both *Chlamydiales* and *C. burnetii.*

Of the ten cases positive for *Chlamydiales*, five showed Cts ≤35 and were further sequenced. Two sequences revealed uncultured *Chlamydiales* and *C. abortus* as the best BLAST hits. The remaining two sequences were of poor quality, with an apparent superposition of sequences, likely due to the presence of more than one member of the *Chlamydiales* order in each sample.

### 3.7 *N. caninum*

Of the 60 brains available for the detection of *N. caninum*, nine were positive by real-time PCR (15%, 95% CI [7.1, 26.6]) (Table 4). One of the cases testing positive for *N. caninum* was also positive for pathogenic *Leptospira* spp., and another was positive for a member of *Chlamydiales.*

### 3.8 Pathological findings

The macroscopic findings for 52 cases and corresponding histological findings for 40 placentas are summarized in Table 5 and Supplementary Table 2, together with the results of comprehensive microbiological analyses. The results of six cases previously reported in Vidal et al. (2017b) are included. The most common macroscopic finding was yellow discoloration of the placental cotyledons, which most often histologically corresponded to moderate to severe necrosis. Malformation was observed in 13 fetuses, eight of which were positive for SBV.

**Table 5:** Overview of the findings of the 52 cases of abortion for which placental tissue was analysed macroscopically and histopathologically and comparison with the results of broad-spectrum bacterial and fungal culture, real-time PCR for *Chlamydiales*, *C. burnetii*, *Leptospira* spp., *N. caninum* and SBV, antigen detection for BVDV and antibody detection for BHV-1 and SBV virus, including the organ where the agent was detected. Samples with a high degree of autolysis were disregarded in the comparison. NC = no changes, P = neutrophilic placentitis, N = necrosis, V = vasculitis, M = mineralization, Ag = antigen, Ab = antibody, BVDB = bovine viral diarrhea virus, BHV-1 = bovine herpesvirus type-1, SBV = Schmallenberg virus, (+) = minimal, + = mild, ++ = moderate, +++ = severe, NT = not tested.

Case ID	Pathological findings					Diagnostic tool			Organ			Comments	Possible etiology
	Macroscopy	Histology				Culture	Real-time PCRs	Ag/Ab detection (BVDV/BHV-1/SBV)	Placenta	Abomasal content	Brain		
		P	N	V	M								
12Ue0622	NC	++	++	++	-	*S. uberis*	*C. burnetii;* Unidentified *Chlamydiales*^a^	Neg/Neg/NT	*S. uberis; C. burnetii; Chlamydiales*	*S. uberis; C. burnetii*	-	*S. uberis* isolated in axenic culture from liver and lung	*S. uberis; C. burnetii*
12Ue0825	NC	++	++	++	+++	*E. coli*	*N. caninum*	Neg/Neg/NT	*E. coli*	*E. coli*	*N. caninum*	*E. coli* isolated in axenic culture from liver and lung. Multifocal necrotizing fetal encephalitis by histopathology.	*N. caninum - E. coli*
12Ue0928	Yellow cotyledons	+++	+++	+	+++	*S. uberis*	Neg	Neg/Neg/NT	*S. uberis*	*S. uberis*	-	*N. caninum* NT	*S. uberis*
12Ue1096	Yellow cotyledons	+	+	+	++	Neg	Neg	Neg/Neg/NT	-	-	-	*Pseudomonas* spp. detected in Vidal et al., (2017b)	Uncultured *Pseudomonas* spp.
12Ue1185	NC	+	+	+	++	Neg	*Leptospira* spp.; *N. caninum*	Neg/Neg/Neg	*Leptospira* spp.	*Leptospira* spp.	*N. caninum*	*-*	*Leptospira* spp. - *N. caninum*
12Ue1228	Fetal malformation	NT				Neg	Neg	Neg/Neg/**Pos**	-	-	-	*N. caninum* NT	SBV
12Ue1503	NC	++	++	++	++	Neg	Uncultured *Chlamydiales*^b^	Neg/Neg/Neg	Uncultured *Chlamydia*	-	-	-	Uncultured *Chlamydia*
12Ue1534	Fetal malformation	NT				Neg	SBV	Neg/Neg/**Pos**	-	-	SBV	-	SBV
12Ue1536	Traumatic diaphragmatic hernia	NT				Neg	Unidentified *Chlamydiales*^c^	Neg/NT/Neg	NA	Unidentified *Chlamydiales*	-	-	Non-infectious cause
13Ue0053	Fetal malformation	NT				Neg	Unidentified *Chlamydiales*^d^	Neg/Neg/Neg	Multiple *Chlamydiales*	NA	-	*N. caninum* NT	Non-infectious cause
13Ue0056	Fetal malformation	NT				Neg	SBV	Neg/Neg/**Pos**	-	-	-	Virus detected in the spleen by PCR; *N. caninum* NT	SBV
13Ue0141	Fetal malformation	NT				Neg	Neg	Neg/NT/**Pos**	-	-	-	-	SBV
13Ue0200	NC	++	++	+	+	Neg	Neg	Neg/Neg/Neg	-	-	-	-	Unclear
13Ue0217	Fetal malformation	(+)	(+)	(+)	(+)	*B. licheniformis*	Neg	Neg/Neg/**Pos**	*B. licheniformis*	*B. licheniformis*	-	-	SBV
13Ue0218	Yellow cotyledons; crumbly consistency	(+)	(+)	(+)	+	Neg	Uncultured *Chlamydiales*^e^	Neg/Neg/**Pos**	Uncultured *Chlamydiales*	-	-	Commensal microbiota detected in Vidal et al., (2017b)	SBV
13Ue0236	NC	NT				Neg	SBV	Neg/Neg/**Pos**	-	SBV	-	*N. caninum* NT	SBV
13Ue0238	Fetal malformation	NT				Neg	Neg	Neg/Neg/**Pos**	-	-	-	Autolytic microbiota detected in Vidal et al., (2017b)	SBV
13Ue0253	Yellow cotyledons	+++	+++	++	++	Neg	*N. caninum*	Neg/Neg/NT	-	-	*N. caninum*	Protozoal cysts in placenta by histopathology	*N. caninum*
13Ue0268	Yellow cotyledons; crumbly consistency	+++	+++	++	+++	Neg	*N. caninum*	Neg/Neg/Neg	-	-	*N. caninum*	Protozoal cysts detected in placenta by histopathology	*N. caninum*
13Ue0274	Fetal malformation	NT				Neg	Neg	Neg/Neg/**Pos**	-	-	-	*N. caninum* NT	SBV
13Ue0348	Yellow cotyledons	++	++	++	+++	*S. pluranimalium*	Neg	Neg/Neg/Neg	*S. pluranimalium*	*S. pluranimalium*	-	-	*S. pluranimalium*
13Ue0448	Fetal malformation	NT				Neg	SBV	Neg/Neg/**Pos**	-	SBV	-	Virus detected in the spleen by PCR; *N. caninum* NT	SBV
13Ue0458	Whyte cotyledons with small hemorrhages	++	++	+	+	Neg	Neg	Neg/Neg/Neg	-	-	-	-	Unclear
13Ue0486	Yellow cotyledons	+	+	+	-	Neg	Neg	Neg/Neg/Neg	-	-	-	-	Unclear
13Ue0526	NC	+	+	(+)	+	Neg	Unidentified *Chlamydiales*^f^, *N. caninum*	Neg/Neg/Neg	Unidentified *Chlamydiales*	-	*N. caninum*	-	*N. caninum*
13Ue0536	Fetal malformation	++	+++	+++	+++	Neg	*C. burnetii*	Neg/Neg/Neg	*C. burnetii*	-	-	-	*C. burnetii*
13Ue0754	NC	+++	+++	+++	+++	Neg	*N. caninum*	Neg/Neg/Neg	-	-		-	*N. caninum*
13Ue0755	NC	+++	+++	+	+	*E. coli*	*N. caninum*	Neg/Neg/Neg	*E. coli*	*E. coli*	*N. caninum*	+++ rods detected in placenta by histopathology; *E. coli* isolated in axenic culture from liver and lung	*E. coli - N. caninum*
13Ue0851	NC	+++	+++	+++	(+)	Neg	Neg	Neg/Neg/Neg	-	-	-	*Enterobacteriaceae, Enterobacteriales* and *Trueperella* detected in Vidal et al., (2017b)	*Enterobacteriaceae, Enterobacteriales* and *Trueperella*
13Ue0858	Diffuse necrosis with a red rim in placenta	++	+++	++	+++	Neg	*C. burnetii*	Neg/Neg/Neg	*C. burnetii*	*C. burnetii*	-	-	*C. burnetii*
13Ue0862	Fetal malformation	NT				Neg	Neg	Neg/Neg/Neg	-	-	-	-	Non-infectious cause
13Ue0974	NC	+++	+++	+++	+++	Neg	Neg	Neg/Neg/Neg	-	-	-	-	Unclear
13Ue1009	Fetal malformation	+++	+++	+	+++	Neg	*C. burnetii; C. abortus*^g^	Neg/Neg/Neg	*C. burnetii; C. abortus*	NA	-	-	*C. burnetii; C. abortus*
13Ue1143	Multifocal red areas in allantochorion	+++	+++	(+)	++	*E. coli*	Neg	Neg/Neg/NT	*E. coli*	*E. coli*	-	*Enterobacteriaceae* detected in Vidal et al., (2017b); *N. caninum* NT	*E. coli*
13Ue1275	NC	+	+	++	+	*S. uberis*	Neg	Neg/Neg/Neg	*S. uberis*	*S. uberis*	-	*Streptococcus* detected in Vidal et al., (2017b)	*S. uberis*
13Ue1360	NC	++	+	-	+++	Neg	*N. caninum*	Neg/Neg/NT	-	-	*N. caninum*	-	*N. caninum*
13Ue1414	NC	+	++	(+)	+++	*E. coli*	*C. burnetii*	Neg/Neg/Neg	*E. coli; C. burnetii*	*E. coli; C. burnetii*	-	-	*C. burnetii*
13Ue1524	NC	+	+	+	+	Neg	*C. burnetii*	Neg/Neg/Neg	*C. burnetii*	*C. burnetii*	-	Trophoblasts with intracytoplasmatic bacteria detected by histopathology	*C. burnetii*
14A0007	NC	NT				Neg	Neg	Neg/Neg/NT	-	-	-	Intrauterine stress	Non-infectious cause
14A0008	Yellow cotyledons	+	+	+	++	Neg	Neg	Neg/NT/NT	-	-	-	-	Unclear
14A0009	NC	(+)	(+)	-	+	Neg	Neg	Neg/Neg/Neg	-	-	-	*N. caninum* NT	Unclear
14A0014	NC	+	+++	+	+	*S. pluranimalium*	Neg	Neg/Neg/Neg	*S. pluranimalium*	*S. pluranimalium*	-	-	*S. pluranimalium*
14A0020	Cotyledons with dark-red discoloration	+	+	+	+	Neg	Neg	Neg/NT/NT	-	-	-	Dam with septicemia	Non-infectious cause
14A0026	Hemorrhagic cotyledons	++	++	+++	-	*C. fetus* subsp. *fetus*	Neg	Neg/NT/NT	*C. fetus* subsp. *fetus*	*C. fetus* subsp. *fetus*	-	-	*C. fetus* sp. f*etus*
14A0027	Yellow cotyledons; crumbly consistency	++	+++	++	++	*S. pluranimalium*	*N. caninum*	NT/Neg/NT	*S. pluranimalium*	*S. pluranimalium*	*N. caninum*	Protozoal cysts detected in placenta by histopathology	*N. caninum; S. pluranimalium*
14A0046	Yellow cotyledons; crumbly consistency	++	++	+	+++	Neg	Neg	Neg/Neg/NT	-	-	-	-	Unclear
14A0051	White cotyledons	++	++	+	+	Neg	Neg	Neg/Neg/NT	-	-	-	-	Unclear
14A0056	NC	++	+	+	-	*S. uberis*	Neg	Neg/Neg/NT	*S. uberis*	-	-	-	*S. uberis*
14A0057	NC	++	++	+	+	Neg	Neg	Neg/Neg/NT	-	-	-	Dam was sick for an extended period	Non-infectious cause
14A0076	Fetal malformation	++	(+)	(+)	+++	Neg	*C. burnetii*	Neg/Neg/**Pos**	*C. burnetii*	-	-	-	SBV
14A0090	Internal bleeding from umbilical arteries	+	(+)	-	(+)	Neg	*C. burnetii*	Neg/Neg/NT	*C. burnetii*	*C. burnetii*	-	-	Non-infectious cause
14A0154	Yellow cotyledons	+	++	++	+++	Neg	Neg	Neg/Neg/NT	-	-	-	-	Unclear

a Not interpretable (multiple peaks).

b Uncultured *Chlamydiales* bacterium clone HE210023biof (99.3% similarity^;^ JX083111).

c Sequencing not possible (Ct > 35).

d Not interpretable (multiple peaks).

e Uncultured *Chlamydiales* bacterium clone 120_13 (95% similarity; KX451030).

f Sequencing not possible (Ct > 35).

g *Chlamydia abortus* (99.3% similarity; Z49871).

The most common histological findings in placentas were neutrophilic inflammation, necrosis, vasculitis and mineralization, mainly affecting the cotyledons. In 27 cases, moderate to severe suppurative and/or necrotizing placentitis was present, 15 cases of which were associated with vasculitis. However, H&E staining revealed no fungal structures. Although occasional pleomorphic fungal hyphae were identified in tissue sections stained with PAS, they were not directly associated with the inflammation, but were intermingled with plant material on the tissue surface.

**Fig. 1:**
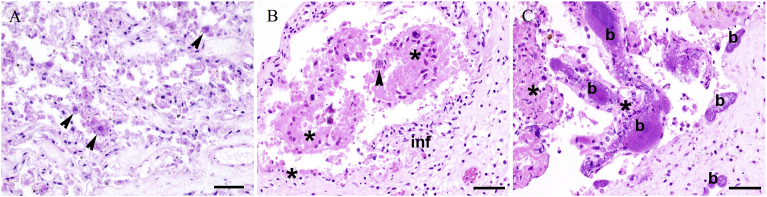
Histopathology of representative placental samples from bovine abortions. (A) Case 13Ue1524, most likely etiology: *Coxiella burnetii*. The epithelium of the placental villi is diffusely necrotic. Few trophoblast cells contain intracytoplasmic bacteria compatible with *C. burnetii* (arrowheads). (B) Case 13Ue0253, most likely etiology: *Neospora caninum*. Moderate suppurative inflammation of the interstitium (inf) and necrosis of the villar epithelium (*). Parasitic cysts containing tachyzoites (arrowhead) are present. (C) Case 13Ue0755, possible etiology: *Escherichia coli*. Large colonies of intravascular coccoid rods (b) expand the lumina of placental vessels. Placental villi show necrosis (*) and moderate suppurative inflammation. DNA of *N. caninum* was also detected in the fetal brain. HE, 400x, bar = 50 μm.

**Fig. 2:**
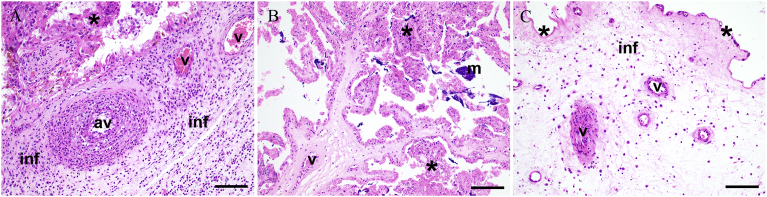
Histopathology of representative placental samples from bovine abortions. (A) Case 14A0026, most likely etiology: *Campylobacter fetus* subsp. *fetus*. The placental interstitium is affected by severe mixed cellular inflammation (inf). The arterial vessels (av) show severe neutrophilic mural infiltration, and the venous vessels (v) present fibrinoid necrosis. Necrotic villar epithelium (*). (B) Case 13Ue0348, most likely etiology: *Streptococcus pluranimalium*. Multifocal necrosis of villi (*) and mild dystrophic mineralization (m). Villar vessels show suppurative vasculitis (v). (C) Case 13Ue1275, most likely etiology: *Streptococcus uberis*. The placenta shows multifocal mild superficial necrosis (*), mild mixed cellular interstitial infiltrate (inf), and marked neutrophilic and necrotizing vasculitis. HE 200x, bar = 100 μm.

At least one possible bacterial or parasitical abortive agent was detected in the 21 cases with histologically evident placentitis (Table 5, Supplementary Table 2). Conversely, in six cases, all analyses showed negative findings, despite the presence of suppurative and/or necrotizing placentitis. Six of eight cases positive for *C. burnetii* exhibited moderate to severe placentitis (Fig. 1A), though prominent vasculitis was evident in only three of these cases. Moreover, one of these three cases was also positive for *C. abortus*. In three of the nine cases positive for *N. caninum* in the brain, protozoal cysts were also visible in the inflamed and necrotic placenta (Fig. 1B). One case positive for *N. caninum* in the brain and in which *E. coli* was isolated in axenic culture from the placenta and fetal organs presented histologically with moderate suppurative and necrotizing placentitis and large intravascular colonies of coccoid rods (Fig. 1C). The only case positive for *C. fetus* subsp. *fetus* showed moderate suppurative and necrotizing placentitis with severe vasculitis and was negative for other abortifacients (Fig. 2A). Four cases positive for *S. uberis* and three cases positive for *S. pluranimalium* presented mild to severe suppurative and necrotizing placentitis; two cases each positive for *S. uberis* or *S. pluranimalium* displayed vasculitis (Fig. 2B and C). The only case positive for *Leptospira* spp. was also positive for *N. caninum*, and presented mild neutrophilic placentitis, necrosis and vasculitis (Table 5).

### 3.9 Overall results

Among the 52 cases summarized in Table 5, a possible bacterial, viral or parasitic etiology was identified in 36 (69.2%, 95% CI [56.7, 81.8]), whereas in seven cases, the abortion was clearly due to non-infectious causes (13.5%, 95% CI [4.2, 22.7]). Among the nine remaining unclear cases (17.3%, 95% CI [7.0, 27.6]), eight presented inflammatory lesions in the placenta compatible with an infectious cause, although an associated pathogenic agent was not identified. Mycotic abortion was not diagnosed in any of the cases. No statistically significant differences were found between the fungal profiles of non-infectious, possibly infectious and unclear cases of abortion or between the fresh and the autolytic abortion material.

## 4 Discussion

Mycotic bovine abortion is commonly sporadic in nature and only affects one or two animals per herd (Austin, 2015). Although a prevalence of bovine mycotic abortion between 2% and 20% is generally estimated (Austin, 2015; Pal, 2015), it appears to be below 2% in Switzerland. In a retrospective study from 1986 to 1995, Reitt et al. (2007) detected fungi involved in abortion by culture in 1.7% cases. One may think that the proportion of mycotic abortion in Switzerland has decreased over time considering two comparable studies including cases from 2003 to 2004 (Borel et al., 2006) and from 2006 to 2010 (Blumer et al., 2011) with a prevalence of mycotic abortion of 1.28%, and 0.29%, respectively. In the present study, ITS2 amplicon sequencing revealed the presence of filamentous fungi and yeasts in the large majority of the abortion material samples. However, a causal link between the presence of fungi and abortion could not be confirmed by histopathology. In previous studies, NGS has been used to reveal infections through the presence of a high number of reads for certain OTUs and by detecting uncultured and difficult-to-grow bacteria, revealing multiple pathogens in the same sample (Hilton et al., 2016; Salzberg et al., 2016; Sabat et al., 2017; Vidal et al., 2017b). Although certain fungi were found at high abundance in our study using amplicon sequencing and were also isolated in culture, they could not be assigned to placental lesions histologically. This suggests that the detected fungi belong to environmental (contaminants) or commensal bovine mycobiota. Abortion tissues are known as material with a high risk of being contaminated through exposure to the genital microbiota and microbes present in the environment (Walker, 2007; Holler, 2012). Sample quality issues are a constant problem in abortion diagnostics and, although superficial contamination can be rinsed away, the sample handling before and time until arrival in the lab are seldom optimal (Holler, 2012). As fungi are ubiquitous in the environment (Kobayashi, 1996), it is important to interpret isolated molecular findings with caution and in conjunction with histopathological results (Walker, 2007; Austin, 2015; Borel et al., 2014). Along these lines, it is not surprising that the most abundant phyla in our study were *Ascomycota* and *Basidiomycota*, the two predominant phyla in different soils and environments (O'Brien et al., 2005; Moll et al., 2016). The genera *Candida* and *Cryptococcus*, which were the most predominant fungal genera found in bovine abortion material, are distributed globally and can be detected in different environments. *Candida* species occur in plant materials and in the digestive and urogenital tracts of animals (Quinn et al., 2011), whereas *Cryptococcus* can be isolated from trees, soil and even fresh water (Spickler, 2013). Unidentified *Capnodiales* include plant and human pathogens, endophytes and epiphytes, with some species being animal parasites (Crous et al., 2009). Some genera were found in multiple cases, accounting for more than 75% of reads assigned such as the genus *Malassezia*, which is part of the skin microbiota of cattle (Zia et al., 2015). In contrast, the three control fetuses were negative for fungi. These findings indicate that (i) aseptic collection of the material during necropsy and/or (ii) the short duration from sample collection to sample processing prevented secondary fungal growth. To clarify the potential of NGS to detect new fungal abortive agents, it would be interesting to apply ITS2 amplicon sequencing in selected cases of abortion that are suspicious for fungi according to histopathology, but with no fungal agent clearly identified by culture. Nonetheless, it is difficult to obtain a representative number of cases for Switzerland due to the low rate of fungal abortion.

The workflow for abortion diagnostics should always include the detection of other abortifacients as well as histopathological analyses to achieve a final diagnosis based on the three criteria: (i) the organism is found in large numbers and/or pure or almost pure culture in the fetal abomasum and/or other tissues; (ii) there is associated inflammation in the fetal tissues and/or membranes; and (iii) tests exclude other common abortigenic agents (Kirkbride, 1990; Borel et al., 2014). To obtain a comprehensive overview of infectious agents involved other than potentially relevant fungi, we thus gathered additional data retrospectively to broaden the spectrum of analyses to include further microbial, molecular and pathological methodologies. Complementary pathological analyses are needed to avoid overinterpretation of mere detection of the opportunistic pathogens (Vidal et al., 2017a). Data from the histopathological examination of fetal tissues was not available to complement the analyses except for one case. Especially for the definitive diagnosis of neosporosis the detection of microscopic lesions in the fetal CNS, heart, and/or liver is needed (Dubey and Schares, 2006). However, many pathologists suggest that placental examination often has a higher diagnostic value than examination of the fetus. This may be due to several reasons including that the underdeveloped fetal immune system may not produce significant lesions, that fetuses often die before lesions appear, and tissues thus present autolysis, and that fetal pathology may be difficult to recognize (Whittier, 2009; Baumgartner, 2015). The isolation of a major abortigenic agent by culture or molecular methods in discordance with compatible lesions – be it lack of lesion or lack of examination – may therefore only point towards a possible causative role. Although all samples were negative for mycotic abortion, we were able to identify a possible cause in 43 of 52 cases (83%) for which data from broad-spectrum analysis were available (Table 5). Previous studies have shown that extended screening of abortive agents increases the diagnosis rate to as high as 56.9%, depending on the analyses applied (Jamaluddin et al., 1996; Khodakaram-Tafti and Ikede, 2005; Corbellini et al., 2006; Reitt et al., 2007; Clothier and Anderson, 2016).

After its first detection in 2011, SBV spread rapidly across Europe, including Switzerland (Hoffmann et al., 2012), where it was detected in bulk tank milk samples and in serum from healthy cows (Balmer et al., 2014, 2015). In a previous study at the national level, no significant differences in fertility were observed between case (SBV-positive) farms and control farms (Wüthrich et al., 2016). This is the first study to include results from SBV testing in a broad-spectrum analysis of bovine abortions in Switzerland. Moreover, fetal malformation, which is the main gross lesion associated with SBV (Baumgartner, 2015), was found in eight of 11 SBV-positive cases. All cases were negative for the other two tested viruses, BHV-1 and BVDV, highlighting the success of the Swiss eradication programs started in 1988 (Ackermann et al., 1990) and 2008 (Presi and Heim, 2010), respectively.

The detection of *N. caninum* in 15% of the cases is in concordance with previous studies carried out in Switzerland, where this parasite has always been diagnosed in more than 16% of studied abortion cases (Fischer et al., 2003; Borel et al., 2006; Blumer et al., 2011). As this pathogen is generally detected in a high percentage of cases and is associated with high economic loss (Estill and Scully, 2015), it should not be overlooked in routine diagnosis. Importantly, the histopathological detection of microscopic lesions in fetal tissues must be taken into account for a definitive diagnosis of neosporosis (Dubey and Schares, 2006). Due to the retrospective approach used in this study such lesions could only be truly confirmed in one case.

A bacterial agent was identified in axenic culture in 24.3%, *Chlamydiales* in 13.5%, *C. burnetii* in 10.8% and *Leptospira* spp. in 1.4% of cases. Among the 52 completely analyzed cases of the total 74, a bacterial agent was identified as the most likely etiological agent in 20 cases. Of these, three cases presented co-infection with multiple bacteria, including *C. burnetii*, *C. abortus*, *S. uberis*, *Enterobacteriaceae, Enterobacteriales* and *Trueperell*a. Furthermore, we documented possible co-infection with *N. caninum* and *E. coli*, *S. pluranimalium* or *Leptospira* spp. in four cases. Unfortunately, except for one case of co-infection with *E. coli*, the suspicion of neosporosis could not be proven due to lack of histology of fetal organs. In Switzerland, *Chlamydiales* and *C. burnetii* have been described as major causes of abortion (Borel et al., 2006; Blumer et al., 2011; Vidal et al., 2017a), whereas *E. coli*, *S. uberis*, *Trueperella* and *Leptospira* spp. play a role in sporadic abortion (Vidal et al., 2017a, b). Co-infections are more likely to occur in countries where the classical abortive agents, such as *Brucella*, do not play a role. This has been previously discussed, e.g. co-infections with *C. abortus*, *Parachlamydia*, *N. caninum* or BVD (Blumer et al., 2011; Schnydrig et al., 2017; Vidal et al. 2017a, b) and investigations are underway to study host-pathogen interaction, e.g. between *C. burnetii* and *C. abortus* (Balk et al., 2017).

Although many agents are known to be opportunistic abortifacients in cattle, the placental lesions associated with these opportunists are rarely described. Fig. 1C depicts the large intravascular *E. coli* colonies in a case of abortion in which *N. caninum* was detected in brain tissue and Fig. 2 lesions in the placenta associated with *S. uberis*, *S. pluranimalium* and *C. fetus* subsp. *fetus*. Although co-infections as a cause of abortion in ruminants have been observed (Blumer et al., 2011; Schnydrig et al., 2017; Vidal et al., 2017a, 2017b), the underlying mechanisms are not yet understood.

The etiological diagnostic rate of cases of abortion is undoubtedly increased with the broad-spectrum diagnostic approach including pathological results to back up laboratory findings. However, this approach is costly and only feasible when both fresh placenta and fetus are available and suitable for investigation. A stepwise procedure with an initial focus on the most important epizootic, zoonotic and recurrent agents of the respective region is likely the most feasible approach for gathering abortion surveillance data on a long-term basis.

## 5 Conclusion

NGS approaches may be useful to sporadically screen for novel emerging agents, including new bacteria and viruses. In contrast to 16S rRNA gene amplicon sequencing, sequencing of the fungal ITS2 region did however not provide any additional information to the broad-spectrum screening. Bearing in mind that a thorough investigation including histopathological analyses is needed to determine the etiology of an abortion, the true clinical significance of the presence of abortive organisms (or their DNA) in bovine abortion samples should not be overestimated.

## Declarations

### Author contribution statement

Sara Vidal: Performed the experiments; Analyzed and interpreted the data; Wrote the paper.

Bernd W. Brandt: Analyzed and interpreted the data; Contributed reagents, materials, analysis tools or data.

Martina Dettwiler: Performed the experiments; Analyzed and interpreted the data; Contributed reagents, materials, analysis tools or data.

Carlos Abril, Jenny Bressan, Gilbert Greub, Caroline F. Frey: Contributed reagents, materials, analysis tools or data.

Vincent Perreten: Conceived and designed the experiments.

Sabrina Rodriguez Campos: Conceived and designed the experiments; Analyzed and interpreted the data; Wrote the paper.

### Funding statement

This was supported by project 1.14.07 of the Swiss Federal Food Safety and Veterinary Office and the University of Bern.

### Competing interest statement

The authors declare no conflict of interest.

### Additional information

No additional information is available for this paper.
